# Irrelevant music: How suprasegmental changes of a melody’s tempo and mode affect the disruptive potential of music on serial recall

**DOI:** 10.3758/s13421-020-01037-1

**Published:** 2020-05-08

**Authors:** Judith Schweppe, Jens Knigge

**Affiliations:** 1 Department of Psychology, University of Erfurt, P.O. Box 900 221, 99105 Erfurt, Germany; 2 Department of Music, Faculty of Education and Arts, Nord University, Levanger, Norway

**Keywords:** Working memory, Irrelevant sound effect, Changing-state, Music, Perceptual organization

## Abstract

On tests of verbal short-term memory, performance declines as a function of auditory distraction. The negative impact of to-be-ignored sound on serial recall is known as the irrelevant sound effect. It can occur with speech, sine tones, and music. Moreover, sound that changes acoustically from one token to the next (i.e., changing-state sound) is more disruptive to serial recall than repetitive, steady-state sound. We tested manipulations that resulted in changes in (higher levels of) perceptual organization for more complex tonal stimuli. Within a trial, the first two bars of a well-known melody were repeated (a) in the exact same manner, (b) with variations only in tempo, (c) with variations only in mode (e.g., Dorian or Phrygian), or (d) with variations in both tempo and mode. Participants serially recalled digits in each of the irrelevant sound conditions as well as in a silent control condition. In Experiment 1a, we tested non-music students and, to investigate whether musical expertise affected the findings, additionally tested students majoring in music in Experiment 1b. Across both samples, recall in the irrelevant sound conditions was significantly poorer than in the silent control condition, but only the tempo variation caused an additional harmful effect. The mode variation did not affect recall performance, in either music or non-music students. These findings indicate that, at least with music, changes are a matter of degree and not every additional variation impairs recall performance.

## Introduction

The auditory system is characterized by its openness to environmental information. This enables the detection of potentially important information even when auditory attention is focused elsewhere. However, it comes at the cost of a high degree of distractibility and susceptibility to interference, the effects of which have been investigated in various domains, for instance, in verbal working memory or in the effects of background music on other cognitive tasks. In tests on verbal working memory, performance declines as a function of auditory distraction (e.g., Colle & Welsh, 1976). Serial recall of a list is poorer when participants are exposed to auditory material that they are supposed to ignore during encoding or during a retention interval (e.g., Miles, Jones, & Madden, 1991). This negative impact of to-be-ignored speech or sound on serial recall of written stimuli was first observed with to-be-ignored spoken language and was thus termed the *irrelevant speech effect* (Salamé & Baddeley, 1982). As it has since then also been found with non-speech sounds, such as sine tones (Jones & Macken, 1993) or instrumental music (e.g., Klatte, Kilcher, & Hellbrück, 1995; Salamé & Baddeley, 2018; Schlittmeier, Weißgerber, Kerber, Fastl, & Hellbrück, 2012), it is currently referred to as the *irrelevant sound effect* (Beaman & Jones, 1998). This irrelevant sound effect is a key finding in research on verbal working memory and is also seen as a benchmark finding in short-term and working memory (Oberauer et al., 2018).

A central characteristic of the irrelevant sound effect is that it is heavily influenced by the acoustic characteristics of the to-be-ignored sound: Sound that changes acoustically from one token to the next (i.e., changing-state sound) is more disruptive to serial recall than repetitive, steady-state sound, which often does not impair recall performance at all (changing state effect, e.g., Jones & Macken, 1993; see also Oberauer et al., 2018). This is the case both for speech as irrelevant sound (e.g., a sequence of different digits vs. repetition of the same digit) and for non-speech sounds (e.g., a sequence of sine tones varying in frequency vs. repetition of the same sine tone; Jones & Macken, 1993).

The irrelevant sound effect in general and the changing-state effect in particular are of interest both for application and for theory testing. Relevant applications include, for instance, the effects of noise in work environments and noise management, the influence of classroom noise on student achievement as well as the potential impact of listening to music while doing homework, studying, or performing other cognitive tasks (e.g., Banbury, Macken, Tremblay, & Jones, 2001; Klatte, Bergström, & Lachmann, 2013). The irrelevant sound effect and the observation that it is not restricted to speech but dependent on changing-state sound has stimulated discussion among several theorists of competing models (e.g., Baddeley & Hitch, 1974; Cowan, 1995; Hughes, Vachon, & Jones, 2005, 2007; Jones & Macken, 1993). In particular, accounts that localize the effects in attention (e.g., Cowan, 1995) versus in perceptual organization (e.g., Jones & Macken, 1993) are currently under discussion.

The embedded-processes model (Cowan, 1995, 1999) attributes the irrelevant sound effect as well as the changing-state effect to attentional capture. Irrelevant sound provokes an orienting response in listeners and thus attracts attention away from the focal task and away from the currently attended representations of the memoranda. Thus, their activation and, in turn, the probability of successful recall, may be reduced (see also Buchner, Bell, Rothermund, & Wentura, 2008). Attentional capture should be more probable with changing-state sound than with steady-state sound, as listeners should habituate to steady-state sound. Thus, the former disrupts performance more strongly than the latter.

In their object-oriented episodic record (O-OER) model, Jones and Macken (1993) explained the changing-state effect such that changes from tone to tone lead to a pre-attentive segmentation of the auditory stream into separate acoustic objects. This goes along with an automatic generation of order cues for the separate objects, which interfere with the order cues required for serial recall. Recently, the O-OER model has been subsumed under a wider competition-for-action (Hughes, Vachon, Hurlstone, Marsh, Macken, & Jones, 2011) or interference-by-process framework (Hughes & Jones, 2005; Jones & Tremblay, 2000). Similarly, these accounts assume that acoustic changes as they are typical for speech yield order cues that conflict with vocal-motor sequence-planning within the focal task. Hence, interference is not due to similarity of the representations but of the processes involved. Crucial predictions from these accounts are that irrelevant sound effects should only occur with changing-state sound and that a central role of serial order processing in the focal task is a key determinant for the changing-state effect.

Many studies have demonstrated that changing-state sound is particularly disruptive to serial recall performance and that changing-state non-speech sound can be as disruptive as irrelevant speech (e.g., Jones & Macken, 1993). These findings are most straightforwardly predicted by the O-OER model. There are, however, also cases in which acoustic variations do not influence serial recall performance. For instance, speech and non-speech sounds varying in intensity do not impair recall more strongly than sounds not varying in intensity (e.g., Ellermeier & Hellbrück, 1998; Tremblay & Jones, 1999). In addition, the relationship between the size of the changes and the size of the changing-state effect is not necessarily linear, as great spectral differences have been found to be less disruptive than smaller ones (Jones, Alford, Bridges, Tremblay, & Macken, 1999). These findings indicate that changes in state cannot simply be specified as mismatches between successive stimuli in terms of any physical characteristics (see also Jones, Beaman, & Macken, 1996; Tremblay & Jones, 1999).

How can these divergent findings be accounted for? According to Tremblay and Jones (1999, p. 1011), patterns of interference of irrelevant sound can “reveal the extent of processing at the preattentive level,” indicating, for instance, that sound level is not encoded. This might imply that changes in sound level are superfluous to object formation and that, thus, variations in loudness are not interpreted as changes in objects’ identities (Tremblay & Jones, 1999). To avoid a circular argument, it would, however, be good to have a priori criteria for object formation and pre-attentive processing (or for what captures attention). Regardless, these findings imply that there are still open questions, such as which acoustic changes lead to changing-state effects in immediate serial recall and under which conditions do greater changes incur greater disruption? (for a similar argument, see Schlittmeier et al., 2008). One aspect that may affect what kind of manipulations influence the size of the irrelevant sound effect is the type of irrelevant sound. A major distinction here concerns speech versus non-speech sound. It is still under discussion whether certain features of speech make it unique as a source of interference or whether non-speech sound can be as disruptive as speech sound as long as it shares important characteristics – that is, whether or not the same principles underlie disruption by irrelevant speech and by irrelevant non-speech sound (LeCompte, Neely, & Wilson, 1997; Neath & Surprenant, 2001; Röer, Bell, & Buchner, 2014a).

When it comes to non-speech irrelevant sound, sequences of sine tones are typically used that are rather limited in their acoustic characteristics as well as in the type of manipulations that one can apply to them. A more complex type of irrelevant sound that people encounter frequently in authentic situations and that allows for systematic manipulations is music. A few studies have investigated the effects of background music on various cognitive tasks from an applied perspective (e.g., Balch, Bowman, & Mohler, 1992; Oakes & North, 2006; for a meta-analysis, see Kämpfe, Sedlmeier, & Renkewitz, 2011). There are also studies that have investigated and demonstrated the effect of irrelevant music on immediate serial recall (e.g., Klatte & Helbrück, 1993; Klatte et al., 1995; Perham & Vizard, 2011; Röer, Bell, & Buchner, 2014b; Salamé & Baddeley, 1989; Schlittmeier, Hellbrück, & Klatte, 2008; Schlittmeier et al., 2012). One central finding of these studies is that vocal as well as instrumental music can induce irrelevant sound effects (e.g., Klatte et al., 1995).

Only a few studies have systematically applied changing-state manipulations to music (Klatte & Helbrück, 1993; Klatte et al., 1995; Röer et al., 2014a; Schlittmeier et al., 2008). Klatte et al. (1995) observed greater disruption for music played staccato compared to music played legato, with both vocal and instrumental music. Staccato music can be considered changing-state sound as it is characterized by brief pauses between notes because they are played with a very short, detached articulation so that the note does not ring out but instead has a very quick release. In contrast, legato music is played in a smooth and flowing manner, without any breaks between notes, and thus represents steady-state sound. Klatte et al.’s (1995) findings thus indicate a changing-state effect based on a musical parameter. Along similar lines, Perham and Sykora (2012) found that both liked and disliked music decreased serial recall performance, but the negative impact was higher for liked music. The authors attributed this difference to less acoustical variation in the disliked music, which was a grindcore metal song in which the individual elements were relatively indiscernible from each other compared to a fast-tempo dance track in which individual musical elements are clearly identified. Röer et al. (2014a) investigated the influence of piano melodies with which participants were familiarized in a passive listening period and that were presented with either an expected but changing-state ending or with an unexpected steady-state ending consisting of a repeated single tone. Thus, the effects of local auditory changes and expectation violations could be contrasted directly. In this case, the expectation violation caused more disruption than the (expected) changing-state ending (see also Parmentier, Elsley, Andrés, & Barceló, 2011).

The latter finding indicates that factors of suprasegmental organization may affect the irrelevant sound effect and that change may also be important as a global property of a sound sequence. It contrasts, however, with other findings. Parmentier and Beaman (2014) manipulated the regularity of inter-stimulus intervals and thus varied whether irrelevant speech (consisting of a sequence of words) was presented in an irregular or a regular rhythm. This rhythm manipulation did not affect serial recall performance as did other manipulations of suprasegmental organization: for instance, whether a sequence of to-be-ignored letters occurred in a fixed and repeated order and was therefore predictable or in random and thus unpredictable order (Jones, Madden, & Miles, 1992). Interestingly, in a combined analysis of their five experiments, Parmentier and Beaman (2014) even found a small reverse effect such that sounds presented with regular timing were unexpectedly more disruptive than sounds with irregular intervals. Based on this, the authors suggested that the irregular timing affected parsing such that irrelevant words presented in close proximity may have been grouped and parsed as two-part tokens. Consequently, the irregular stream would have resulted in fewer between-unit transitions and thus a lower word dose (Bridges & Jones, 1996). On the other hand, the grouped words would include more changes within an auditory object than the single words in the regular stream. Thus, it may be that changes in state are not only relevant between auditory objects but also within objects, and that “the difference between changing-state and steady-state is a matter of degree rather than kind” (Parmentier & Beaman, 2014, p. 36).

Röer et al.’s (2014a) findings further indicate that effects of suprasegmental organization of the irrelevant sound on memory for serial order may be overlooked with irrelevant sound sequences made up of very simple stimuli but may be more easily detectable when the irrelevant sound consists of music (or of more complex and inherently structured speech such as sentences; Röer et al., 2014a, Exp. 2). In our study, we wanted to follow up on these findings by applying two manipulations to music as irrelevant sound, both of which concerned larger units and thus changes in suprasegmental organization. These are not classic changing-state manipulations, as they do not concern changes from tone to tone, but rather constitute suprasegmental changes and thus augment the concept of changing-state to higher levels of perceptual organization.

Fundamental musical parameters that are central for music perception and that could therefore influence the irrelevant sound effect are, for instance, tempo (e.g., Fraisse, 1982), mode (e.g., Bigand & Poulin-Charronnat, 2016), instrumentation (e.g., McAdams & Giordano, 2009), and articulation (e.g., Schmuckler, 2009). We focused on two of these – mode and tempo, as both can be related to Parmentier and Beaman’s (2014) rhythm manipulation.

Western music can be characterized by means of its hierarchical organization in which tonal hierarchy is a central feature (Bigand & Poulin-Charronnat, 2016). That is to say, in a given melody not every pitch event has the same importance because pitch events are heard in a hierarchy of relative importance (e.g., Lerdahl & Jackendoff, 1983). There has been a large amount of theoretical and empirical work about the organization in the tonal pitch structure and how this organization influences music perception. Two of the most important frameworks are Lerdahl’s (2001) “tonal pitch space theory” and Bharucha’s (1987) “model of musical activation.” For our context, it was crucial that changes in mode (e.g., major vs. minor) result in a mental reorganization of the hierarchical values of tones. Furthermore, these changes in cognitive reference points strongly affect how a music piece is perceived (Bigand & Poulin-Charronnat, 2016; Schmuckler, 2016). Therefore, it is plausible to assume that mode changes would promote segmentation of an auditory stream of music into separate objects.

Also, tempo is a fundamental musical parameter that is important for music perception. In fact, numerous studies have shown that tempo is the most relevant factor regarding emotional expression (e.g., Gabrielsson, 2016). Faster tempi usually cause associations with expressions of activity, happiness, potency, anger, uneasiness, and so on. However, slower tempi may be associated with expressions such as calmness, peace, sadness, and boredom, among others. Of course, the association with different expressions is not only dependent on tempo but on other structural factors as well. However, Gabrielsson (2016) pointed out that in terms of the valence-arousal model, fast tempi are generally associated with higher activation and slow tempi with lower activation; both of them may be associated with either positive or negative valence. Furthermore, there have been relevant findings regarding tempo sensitivity and spontaneous tempo (e.g., Fraisse, 1982; McAuley, 2010). Extensive experimental research has suggested that there is a preferred tempo range in which human perception is especially sensitive to rhythmical pulsation (Fischinger & Kopiez, 2008). Most recent theories of rhythm perception define this range between 500 and 600 ms (= 120–100 bpm) for inter-onset intervals (e.g., McAuley, 2010; Parncutt, 1994). Changes in tempi that lay within and beyond this range should thus promote acoustic segmentation.

In our study, we presented four repetitions of a brief musical piece that was well known to our participants as irrelevant sound. This was meant to allow for changes to be easily noticeable without a familiarization phase. To this end, we used the first two bars of an instrumental version (sampled piano sound) of a well-known German nursery song (“Alle meine Entchen” [“All my Ducklings”]). Within this auditory stream of irrelevant music, we manipulated whether or not the tempo within each segment varied between the four repetitions and whether the segments were presented in the same mode or in four different modes. We expect variations in both tempo and mode to impair serial recall performance.

## Experiment 1a

The experiment was based on a 2 × 2 × 9 design with tempo changes (yes vs. no) and mode changes (yes vs. no) in the irrelevant sound as well as serial position manipulated within participants. Additionally, a silent control condition was included. The percentage of correct serial recall performance served as the dependent variable. Recalling an item in its original serial position constituted a correct response.

### Method

#### Participants

Thirty-one musical laypersons, who were non-music students at the University of Erfurt and native speakers of German, participated in the experiment in exchange for course credit. One participant did not complete the experiment due to a technical error and thus had to be excluded.

#### Materials and procedure

The memoranda were lists of the digits 1 to 9 presented in random order, with each item presented visually on a screen for 800 ms with an inter-stimulus interval of 200 ms. The presentation of each list was followed by a 10-s retention interval. Finally, the word “Wiedergabe” (recall) appeared on the screen to signal the start of written (typed) recall.

**Fig. 1:**
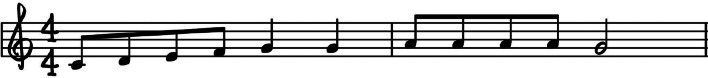
Original version (C major) of the irrelevant sound melody

The irrelevant sound consisted of an instrumental version (piano sound; software: *Logic Pro X*, piano sample: *Toontrack EZ Keys*) of the first two bars of “Alle meine Entchen” (see Fig. 1), which is one of the best-known German nursery songs.

As this melody was well known to our participants, changes were expected to be easily detectable. The two-bar sequence repeated four times per trial, which covered the stimulus presentation and the retention phase. In the condition without any changes, the sequence was repeated in the exact same manner in C major at 107 bpm (with no pauses/silences between the repetition of the melodies). While 107 bpm lies within the preferred tempo range for perception (see above), in the trials with a tempo change, we presented the sequence outside this range with a varying tempo. The two-bar sequence was presented at 60, 80, 122, and 160 bpm in a predetermined random order in each trial (resulting in the same overall sound duration as in the conditions without tempo changes). When a mode change was included, each sequence was presented in a different mode, sampled per trial without replacement from eight different modes (C major, Dorian, Phrygian, Æolian, Lydian, Locrian, whole-tone scale, and semitone-tone-scale; the difference between these modes consists only in the positioning of semitone and whole-tone steps. C was always the keynote; see Appendix). In the conditions without a mode change, the entire sequence was played in C major. Prior to the experiment, the melody sequences were generated as midi data with the software *Logic Pro X* and exported as wav-files with piano sound (*Toontrack EZ Keys*). All irrelevant sound stimuli can be accessed via the Open Science Framework (OSF) at https://osf.io/5rmcy/. The melody sequences were presented via headphones.

After a practice phase consisting of three trials, each participant performed 75 experimental trials: 15 each in the silent control, no change, mode change, tempo change, and mode plus tempo change condition, varied on a trial-by-trial basis. The order of the trials was randomized prior to the experiment and was the same for all participants. The experiment was programmed in Psychopy (Peirce, 2007) and lasted approximately 30 min.

### Results

**Fig. 2:**
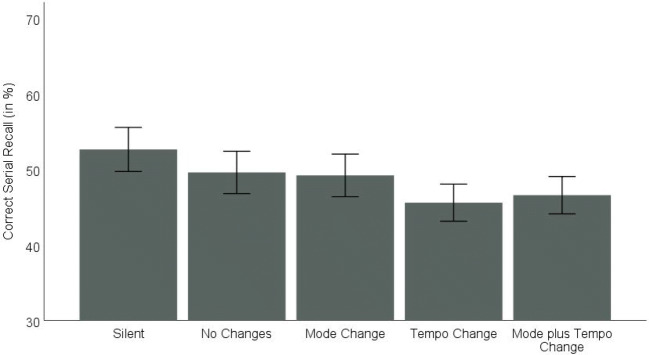
Correct serial recall (in %) in Experiment 1a as a function of the irrelevant sound condition (silent vs. no changes vs. mode change vs. tempo change vs. mode plus tempo change). Error bars indicate standard errors

The data set is available via the OSF at https://osf.io/5rmcy/. The means and standard errors per irrelevant sound condition are displayed in Fig. 2.

We ran two types of analyses: first, a two-way repeated-measures ANOVA with the factors irrelevant sound (silent vs. no changes vs. mode change vs. tempo change vs. mode plus tempo change) and serial position (1 to 9), and, second, a three-factorial repeated-measures ANOVA on the conditions with irrelevant sound only, with the factors tempo change (yes vs. no), mode change (yes vs. no), and serial position (1 to 9).

The ANOVA with irrelevant sound and serial position revealed large significant main effects for both irrelevant sound, *F*(4,116) = 6.55, *p* < .001, η^2^_p_ = .184, and serial position, *F*(8,232) = 86.37, *p* < .001, η^2^_p_ = .749, while the interaction did not reach significance *F*(32,928) = 1.11, *p* = .309, η^2^_p_ = .037. Planned comparisons indicated significantly better recall performance in the silent condition than in the conditions with irrelevant sound combined (53% vs. 48%), *F*(1,29) = 14.62, *p* < .001, η^2^_p_ =.335. Compared solely to the sound condition with no changes, the advantage for the silent condition failed to reach significance (53% vs. 50%), *F*(1,29) = 3.80, *p* = .06, η^2^_p_ =.116.

The ANOVA on the irrelevant sound conditions with the factors tempo change, mode change, and serial position revealed large significant main effects for tempo change, *F*(1,29) = 5.23, *p* = .03, η^2^_p_ = .153, and serial position, *F*(8,232) = 82.09, *p* < .001, η^2^_p_ = .739. When the tempo of the musical sequences did not vary during a trial, performance was superior (49%) compared to the trials with a tempo change (46%). In contrast, mode changes did not affect serial recall performance (48% without vs. 48% with mode changes), *F*(1,29) = 0.01, *p* = .938, η^2^_p_ < .001. Moreover, none of the interactions reached significance (tempo change × mode change: *F*(1,29) = 0.63, *p* = .434, η^2^_p_ = .021; tempo change × serial position: *F*(8,232) = 1.09, *p* = .372, η^2^_p_ = .036; mode change × serial position: *F*(8,232) = 0.35, *p* = .944, η^2^_p_ = .012; tempo change × mode change × serial position: *F*(8,232) = 0.93, *p* = .495, η^2^_p_ = .031).

To test whether the non-significant effect for mode changes can be interpreted as evidence in favor of the null hypothesis, we additionally conducted a Bayesian repeated-measures ANOVA (using JASP 0.9.2, JASP Team, 2019; with default prior scales and serial position included in the null model). With a BF_01_ of 13.699 the data provided strong evidence in favor of the null hypothesis.

### Discussion

Presenting repetitions of a well-known melody resulted in an irrelevant sound effect as was the case with other musical stimuli in prior studies. However, even the difference between the silent condition and the condition with no variations in the four repetitions of the two bars fell just short of reaching significance (with medium effect size). Thus, our “no-changes” sound may be different from typical steady-state sound as it did include changes from tone to tone, albeit not from part to part. This is in line with Parmentier and Beaman’s (2014, p. 36) observation that “changes also occur within objects, so the difference between changing-state and steady-state is a matter of degree rather than kind.”

To investigate whether changes in state that concern larger units additionally harm performance, we manipulated the tempo and mode (same or changed) during the repetition of the brief musical piece. While the tempo variation did indeed cause a suprasegmental changing-state effect, the mode variation did not. The effect of tempo changes contrasts with previous findings in which changes in suprasegmental organization did not cause additional harm. Such changes included speech presented in a regular versus an irregular rhythm (Parmentier & Beaman, 2014) or speech presented in a predictable versus an unpredictable order (Jones et al., 1992). However, even though our tempo manipulation resembled Parmentier and Beaman’s (2014) rhythm manipulation in that pauses between single elements (tones and words, respectively) were either of equal or of varying length, it also differed from previous manipulations of suprasegmental organization in several ways. In our study, these changes in pauses affected larger units as they changed only from repetition to repetition. In addition, the tempo manipulation affected the length of the tones as well as the pauses. Put differently, if each two-bar piece is considered as one stimulus, the rhythm change affected the transitions between stimuli while the tempo change (also) affected transitions within a stimulus.

In contrast to the effect of tempo changes, there was no effect of changing modes at all. The finding that performance did not vary between the trials in which all four repetitions were played in the same mode and those in which the mode changed from repetition to repetition corroborates previous findings that not every acoustic variation impairs serial recall performance, in particular when the change concerns larger units. The rationale of the changing-state hypothesis was that listening to changing-state sound promotes segmentation of the acoustic stream into different objects. If the same logic applies to suprasegmental changes, the null effect may imply that mode changes in unattended sound do not bring about such a pre-attentive segmentation into auditory objects. However, in the music that non-experts typically listen to, mode changes are rare and should thus be a strong signal for auditory segmentation. Such a post hoc interpretation is therefore problematic, and it would be useful to be able to determine whether or not an acoustic change promotes segmentation, independent of its effect on serial recall.

Another reason why mode changes, unlike tempo changes, did not impair performance might be that our participants were musical laypersons and that acoustic segmentation based on changing modes is more likely among participants who are more experienced with music and with different modes. Ear training is an important part of any music study program, and for ear training, the perception and distinction of different tonal modes are crucial. It is therefore possible that musical expertise influences pre-attentive acoustic segmentation. In addition, it could also influence the degree to which a mode change in an unattended stream of music captures participants’ attention. Hence, we assume that with participants who have more experience with music – and especially with analytical listening based on ear training – both tempo changes and mode changes should decrease serial recall performance compared to a condition in which a melody is repeated in the exact same manner. To test this assumption, we replicated Experiment 1a with music students at two universities of music to analyze the data from this sample in combination with the data from Experiment 1a.

## Experiment 1b

The design of Experiment 1b was analogous to that of Experiment 1a with tempo changes (yes vs. no) and mode changes (yes vs. no) in the irrelevant sound as well as serial position (1 to 9) manipulated within participants with an additional silent control condition included. Again, serial recall performance served as the dependent variable. When combining the two samples, the design includes an additional quasi-experimental variable musical expertise (non-music students vs. music students).

### Method

#### Participants

In addition to the 30 non-music students who participated in Experiment 1a, 30 students who majored in music and were native speakers of German participated in exchange for a small honorarium: 18 of them at the Lübeck University of Music (Musikhochschule Lübeck, MHL) and 12 at the State University of Music and the Performing Arts Stuttgart (Staatliche Hochschule für Musik und Darstellende Kunst Stuttgart, HMDK). Three participants had to be excluded due to technical errors.

#### Materials and procedure

In order to allow for a joint analysis with the data from Experiment 1a, materials and procedure were the same as in Experiment 1a except that the experiment was carried out at the MHL and the HMDK.

### Results

**Fig. 3:**
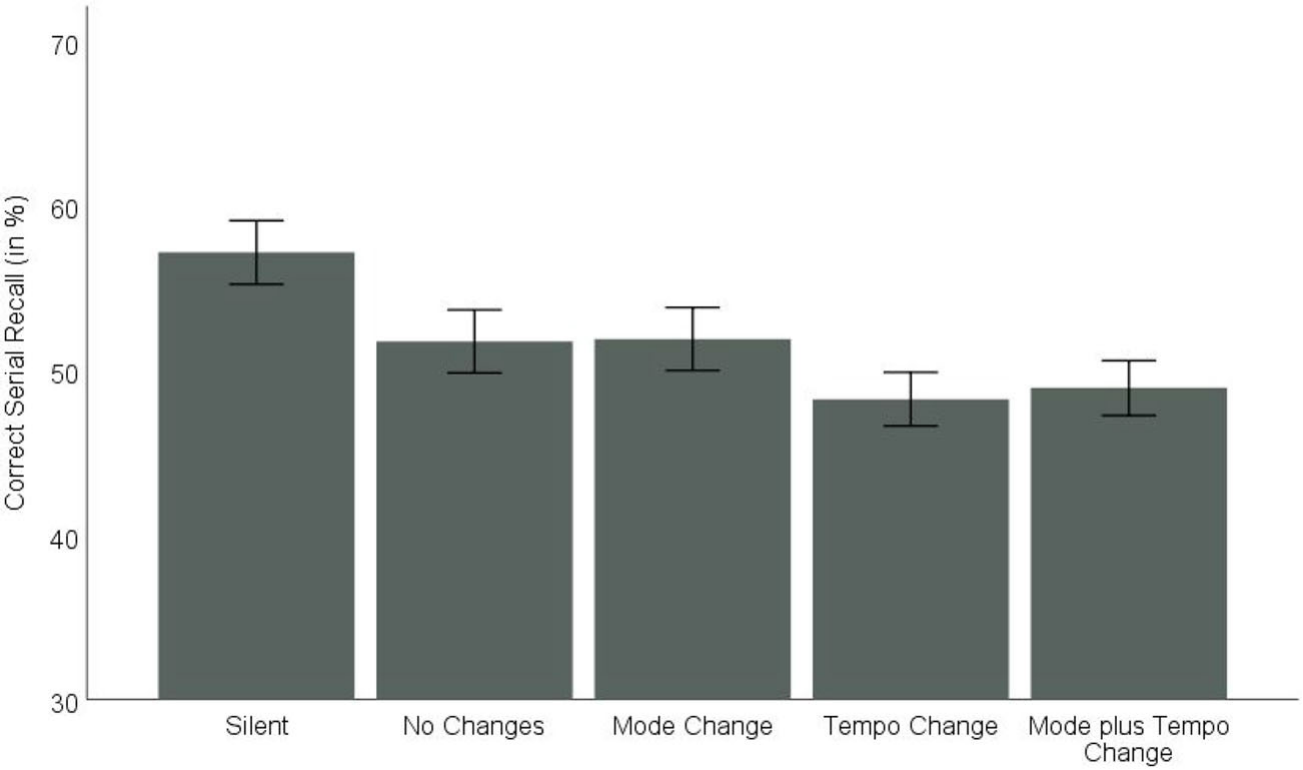
Correct serial recall (in %) in Experiments 1a and 1b as a function of the irrelevant sound condition (silent vs. no changes vs. mode change vs. tempo change vs. mode plus tempo change) collapsed over musical expertise. Error bars indicate standard errors

The data set is available via the OSF at https://osf.io/5rmcy/. Means and standard errors per irrelevant sound condition are displayed in Fig. 3.

In order to test whether our irrelevant sound manipulations affected non-music and music students differently, we conducted two mixed ANOVAs: (1) a three-way ANOVA with the quasi-experimental between-participant factor musical expertise (non-music student sample from Experiment 1a vs. music student sample from Experiment 1b) and the within-participant factors irrelevant sound (silent vs. no changes vs. mode change vs. tempo change vs. mode plus tempo change) and serial position (1 to 9); and (2) a four-way ANOVA on the conditions with irrelevant sound only with musical expertise as a between-participant factor (non-music student sample from Experiment 1a vs. music student sample from Experiment 1b) and tempo change (yes vs. no), mode change (yes vs. no), and serial position (1 to 9) as within-participant factors.

**Fig. 4:**
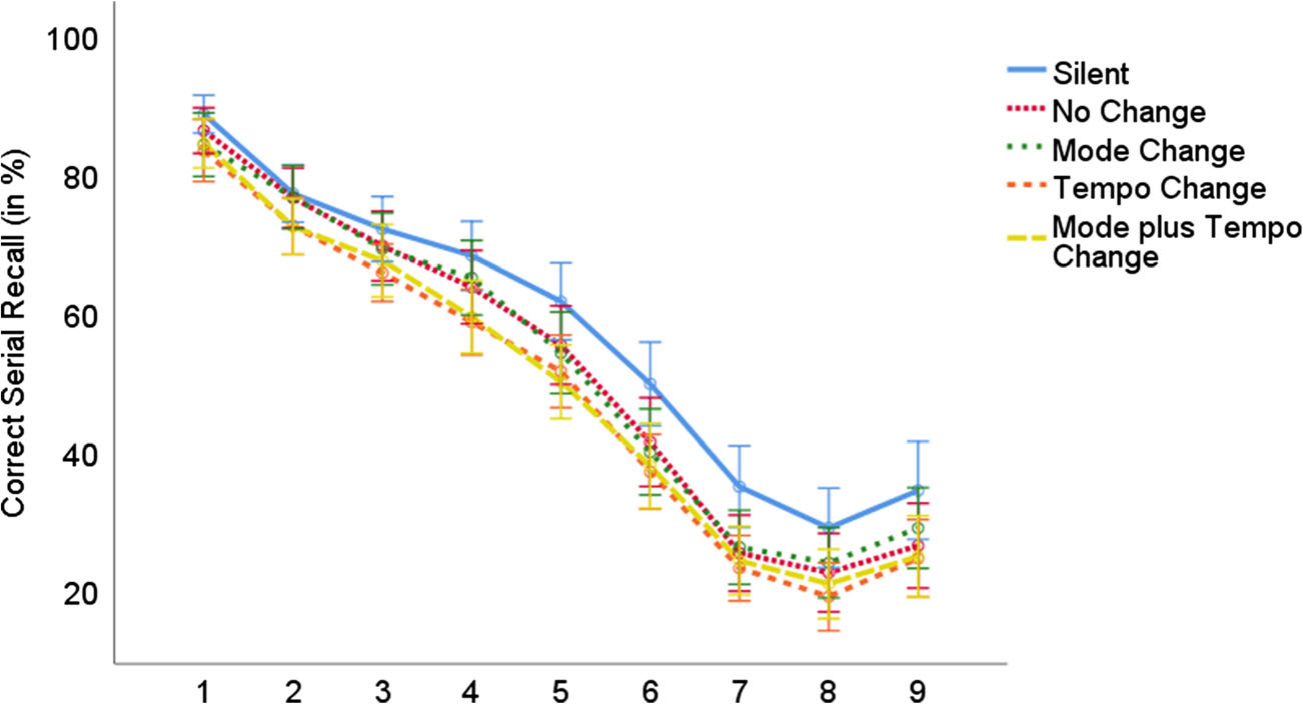
Correct serial recall (in %) in Experiments 1a and 1b as a function of the irrelevant sound condition (silent vs. no changes vs. mode change vs. tempo change vs. mode plus tempo change) and serial position (1 to 9) collapsed over musical expertise. Error bars indicate standard errors

1. The joint ANOVA with the factors musical expertise, irrelevant sound, and serial position revealed large significant main effects for both irrelevant sound, *F*(4,220) = 16.21, *p* < .001, η^2^_p_ = .228, and serial position, *F*(8,440) = 203.41, *p* < .001, η^2^_p_ = .787. According to planned comparisons, serial recall performance was significantly better in the silent condition than in the conditions with irrelevant sound combined (57% vs. 50%), *F*(1,55) = 40.56, *p* < .001, η^2^_p_ =.424. Moreover, there was a significant difference between the silent condition and the sound condition without additional changes (57% vs. 52%), *F*(1,55) = 18.30, *p* < .001, η^2^_p_ = .250. In contrast, the main effect for musical expertise did not reach significance, *F*(1,55) = 3.08; *p* = .085; η^2^_p_ = .053, although there was a descriptive recall advantage for music students (54%) compared to non-music students (49%). The two-way interaction between irrelevant sound and serial position reached significance, *F*(32,1760) = 1.52, *p* = .032, η^2^_p_ = .027. When analyzed separately for each position, the difference between the silent control condition and the irrelevant sound conditions did not reach significance at Position 2 and it generally increased as the list progressed (see also Fig. 4). However, musical expertise did not interact with either irrelevant sound, *F*(4,220) = 1.19, *p* = .316, η^2^_p_ = .021, or serial position, *F*(8,440) = 0.61, *p* = .769, η^2^_p_ = .011. The three-way interaction between musical expertise, irrelevant sound, and serial position did not reach significance either, *F*(32,1760) = 1.38, *p* = .079, η^2^_p_ = .024.

(2) The joint ANOVA on the irrelevant sound conditions with the factors musical expertise, tempo change, mode change, and serial position revealed a large significant main effect for tempo change, *F*(1,55) = 9.58, *p* = .003, η^2^_p_ = .148. Performance was superior when the tempo of the musical sequences did not vary during a trial (52%) compared to the trials with a tempo change (49%). Again, neither mode changes (50% without vs. 50% with mode changes), *F*(1,55) = 0.37, *p* = .547, η^2^_p_ = .007, nor musical expertise affected serial recall performance (48% for non-music students vs. 53% for music students), *F*(1,55) = 2.28, *p* = .137, η^2^_p_ = .040. Moreover, mode change and musical expertise did not interact, *F*(1,55) = 0.03, *p* = .868, η^2^_p_ < .001. We again conducted a Bayesian repeated-measures ANOVA (using JASP 0.9.2, JASP Team, 2019; with default prior scales and “serial position” included in the null model). For mode changes, a BF_01_ of 16.949 provided strong evidence in favor of the null hypothesis, while a BF_01_ of 1.639 provided only inconclusive evidence for the null hypothesis regarding the influence of musical expertise. With respect to the interaction between the two factors (with “serial position,” “tempo,” “mode,” and “musical expertise” included in the null model), however, the data again strongly favor the null hypothesis (BF_01_ = 13.889). A large significant main effect again showed up for serial position, *F*(8,440) = 198.85, *p* < .001, η^2^_p_ = .783. As can be seen in Fig. 4, performance strongly decreased from the first to the penultimate position with a small recency effect. However, serial position interacted neither with tempo change, *F*(8,440) = 0.93, *p* = .491, η^2^_p_ = .017, nor with mode change, *F*(8,440) = 0.66, *p* = .724, η^2^_p_ = .012. Figure 4 indicates that the small difference between the conditions with and without a tempo change was relatively stable throughout the position curve. Even at the final three positions where performance was by far the lowest, there was a 2% difference (25% without vs. 23% with tempo change). Similarly stable across serial positions was the overlap between the conditions with and without mode changes. In addition, none of the other interactions reached significance (musical expertise × tempo change: *F*(1,55) = 0.01, *p* = .942, η^2^_p_ < .001; musical expertise × serial position: *F*(8,440) = 0.83, *p* = .580, η^2^_p_ = .015; tempo change × mode change: *F*(1,55) = 0.13, *p* = .722, η^2^_p_ = .002; musical expertise × tempo change × mode change: *F*(1,55) = 0.34, *p* = .565, η^2^_p_ = .006; musical expertise × tempo change × serial position: *F*(8,440) = 1.11, *p* = .353, η^2^_p_ = .020; musical expertise × mode change × serial position: *F*(8,440) = 1.18, *p* = .311, η^2^_p_ = .021; musical expertise × tempo change × mode change × serial position: *F*(8,440) = 1.37, *p* = .208, η^2^_p_ = .025).

### Discussion

Including an additional sample of students who majored in music revealed no significant differences between participants differing in musical expertise. Irrespective of participants’ background in music education, repeated exposition to the instrumental version of the first two bars of “Alle meine Entchen” (“All my Ducklings”) resulted in an irrelevant sound effect, and changes in tempo additionally impaired serial recall performance, while changes in mode did not. Thus, contrary to our expectations, more experience with – and a formal education in – music did not result in mode changes affecting serial order processing of the memoranda. We assumed that mode is a relevant category for musicians because of their experience with different musical styles and especially because of their expertise regarding ear training. So, our assumption was that our participants would detect the changes in mode automatically. In the *General discussion*, we address potential reasons for the fact that not even in participants with more experience in music did mode changes affect serial recall performance.

## General discussion

We investigated whether acoustic changes between four repetitions of a background melody affected verbal serial recall. We manipulated whether or not the tempo and the mode of the melody changed within one trial. Irrespective of the musical expertise of the participants, even the condition in which the melody was repeated in exactly the same manner led to an irrelevant sound effect compared to a silent condition. On top of that, only a change in tempo resulted in poorer recall performance compared to the repeated melody, while mode changes had no effects, also irrespective of participants’ background in music education.

Thus, whether the tempo of a musical piece stayed the same or changed affected serial recall performance. This manipulation resulted in a descriptively small (only 3% difference in recall performance) but consistent effect (with a large effect size). In terms of the O-OER model (Jones & Macken, 1993) and, more generally, interference-by-process (Hughes & Jones, 2005), this can mean that tempo promotes automatic segmentation into separate acoustic objects. Based on this, order cues are generated that interfere with the serial ordering of the memoranda. Alternatively, this effect could be due to the changes in tempo capturing participants’ attention (e.g., Cowan, 1995).

Not only was the tempo change effect rather small, but it was not larger than the difference between the silent condition and the condition with unchanged repetitions. However, it is worth noting that our “unchanged” sound changed more than the typical steady-state sound in studies with sine tones or speech. In our study, it was not a single tone that was repeated without changes but a sequence of eleven tones. This was in itself already a changing-state condition. The fact that tempo changes reduced recall performance only slightly could also be due to the rather small dose – in each trial, only four renditions of the melody were played. Previous studies have demonstrated that a larger token dose in the irrelevant sound increases its negative impact (e.g., Campbell, Beaman, & Berry, 2002). Nonetheless, the additional effect of the tempo manipulation demonstrates that it is possible to find graded effects of changes.

Another noticeable aspect of our findings is that participants’ serial recall performance even in the silent condition was rather low (57% correct across both experiments and serial positions). Serial position curves indicate that there is a steep decrease after position 6 with performance as low as 19–29% at positions 7 and 8 and also only a small recency effect. Nonetheless, the serial position curves also indicate that the lack of a mode effect is not due to performance being at floor already in the control condition as both the presence of a tempo effect and the absence of a mode effect were relatively stable throughout the position curve.

The tempo change effect in our study was also particular in that the manipulation did not mainly concern local changes from tone to tone but rather from one two-bar piece to another and therefore tapped into suprasegmental organization. This finding goes beyond previous studies in which the pauses between words making up the irrelevant sound were regular or irregular, a manipulation that did not influence recall performance (Parmentier & Beaman, 2014). The difference may be because our tempo manipulation did not only concern the length of the pauses but also that of the tones. Therefore, it might be interesting to manipulate the articulation rate as well as the lengths of pauses in irrelevant speech to see whether that suffices to find a tempo change effect even with speech. Apart from this, the differing effects may also be due to the type of irrelevant sound such that rhythm or tempo manipulations in music are perceived differently from rhythm or tempo manipulations in speech. These findings confirm that one cannot easily generalize from studies using sine tones or unrelated words as irrelevant sound to studies using more complex and authentic background “noise” such as music. Another way in which the tempo manipulation may have affected recall performance is by interfering with participants’ rehearsal rhythm. While participants could have rehearsed at a regular tempo when the irrelevant music did not change in tempo, the tempo changes could have interfered with this process.^1^ Given that the rhythm of irrelevant speech did not affect performance in Beaman and Parmentier’s (2014) study, this would again imply that tempo changes in (irrelevant) music and speech are perceived differently.

The findings further indicate that changes in mode as another type of change from segment to segment do not additionally impair serial recall performance, not even in students who majored in music. Why was this the case? We had assumed that mode changes would promote segmentation of an auditory stream of music into separate objects because changes in mode resulted in a mental reorganization of the hierarchical values of tones (Bigand & Poulin-Charronnat, 2016; Schmuckler, 2016). In addition, changes in mode may have captured attention, at least for the music students, which also should have impaired performance. Not only did the modes deviate from one another but also from typical renditions of “Alle meine Entchen” in C major. The mode change condition thus also did not meet expectations based on prior experience with the melody. However, certain aspects of the mode manipulation may have reduced its impact. The modes we used were primarily different church modes, which even the music students might not have ample perceptual experience with. In addition, the actual tone changes between the modes were sometimes minimal (i.e., there is only one different tone between the original and the Dorian version, but four different tones in the Locrian version). The changes from one mode to another were thus rather gradual and smaller than the staccato/legato comparison applied by Klatte et al. (1995). Moreover, we have investigated mode only as the basis for a melody (with only six different tones) but not for the harmonic structure of accompaniment. Since a mode applied to a harmonic accompaniment could be represented in total, its variation might have a stronger impact.

Thus, both the negative impact of (this type of) music as irrelevant sound and the additional effects of the suprasegmental change manipulations were similar for participants with differing levels of musical expertise. In other words, there was no top-down effect of expertise, which we had assumed to operate at the level of pre-attentive acoustic segmentation or of attentional capture. A drawback in our quasi-experimental variation of musical expertise is that we distinguished the sample solely on the basis of whether or not they were studying music, which is undeniably rather coarse. As we did not include an explicit measure of musical experience, we do not know whether our non-expert sample were experienced music performers and/or listeners outside their studies. The difference between the two samples may thus have been smaller than intended. However, even if this were the case, it would not help explain the null effect of mode changes and the null effect of musical expertise thereon. We had additionally collected data from music students because we had hypothesized that the unexpected null effect of mode changes might be due to our original sample of musical laypersons being too inexperienced with these types of musical modes. Consequently, the problems with the distinction based on university subjects alone do not alter the implication that even participants with some experience in music are not affected by mode changes in irrelevant background music. Nonetheless, it would be insightful to include a finer measure of musical experience in future studies (e.g., the Goldsmiths Musical Sophistication Index; Müllensiefen, Gingras, Musil, & Stewart, 2014; Schaal, Bauer, & Müllensiefen, 2014). This could also shed further light on potential differences in overall performance, regarding which the current data are indecisive.

The standard theories that account for the irrelevant sound effect and the changing-state effect cannot straightforwardly explain the discrepancy in the effects of the tempo and the mode manipulation. To explain why a tempo change should promote segmentation into different objects more strongly than a mode change, further specifications of acoustic and cognitive processing of music are necessary. Further insights could come from variations of the focal task such that it either requires seriation (e.g., a serial recall task) or does not require seriation (e.g., a missing item task). These task variations allow for distinguishing attentional capture and order interference as the bases for effects of changes in irrelevant sound (Hughes et al., 2005, 2007) and could also shed further light on our effects of suprasegmental changes. So far, our studies indicate that music as irrelevant sound provides a rich basis for investigating irrelevant sound effects and, in particular, for investigating gradual differences between sounds that include different degrees of variation.

## Figures and Tables

**Figure:**
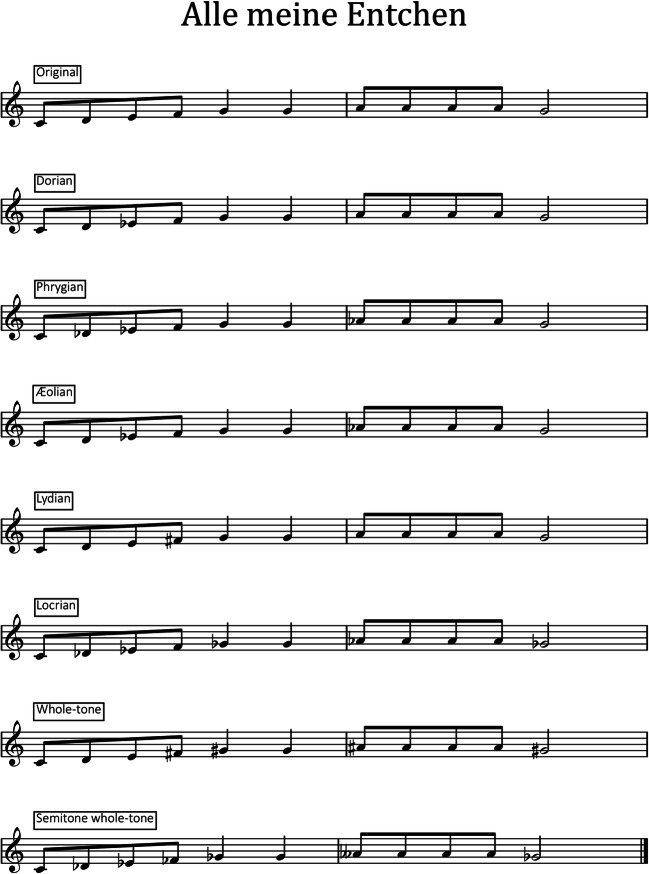
Fig. 5

## Footnotes

1 We thank an anonymous reviewer for this suggestion.
